# Nitric Oxide Alleviated High Salt–Induced Cardiomyocyte Apoptosis and Autophagy Independent of Blood Pressure in Rats

**DOI:** 10.3389/fcell.2021.646575

**Published:** 2021-04-29

**Authors:** Yong Li, Xiaoguang Wu, Yukang Mao, Chi Liu, Yiting Wu, Junzhe Tang, Kun Zhao, Peng Li

**Affiliations:** ^1^Department of Cardiology, The First Affiliated Hospital of Nanjing Medical University, Nanjing, China; ^2^Department of Cardiology, The Affiliated Hospital of Xuzhou Medical University, Xuzhou, China; ^3^The First School of Clinical Medicine, Nanjing Medical University, Nanjing, China

**Keywords:** high-salt diet, cardiomyocytes, apoptosis, autophagy, nitric oxide, sodium nitroprusside

## Abstract

The present study aimed to explore whether high-salt diet (HSD) could cause cardiac damage independent of blood pressure, and whether nitric oxide (NO) could alleviate high-salt–induced cardiomyocyte apoptosis and autophagy in rats. The rats received 8% HSD *in vivo.* H9C2 cells or primary neonatal rat cardiomyocytes (NRCM) were treated with sodium chloride (NaCl) *in vitro*. The levels of cleaved-caspase 3/caspase 3, cleaved-caspase 8/caspase 8, Bax/Bcl2, LC3 II/LC3 I, Beclin-1 and autophagy related 7 (ATG7) were increased in the heart of HSD rats with hypertension (HTN), and in hypertension-prone (HP) and hypertension-resistant (HR) rats. Middle and high doses (50 and 100 mM) of NaCl increased the level of cleaved-caspase 3/caspase 3, cleaved-caspase 8/caspase 8, Bax/Bcl2, LC3 II/LC3 I, Beclin-1, and ATG7 in H9C2 cells and NRCM. The endothelial NO synthase (eNOS) level was increased, but p-eNOS level was reduced in the heart of HSD rats and H9C2 cells treated with 100 mM NaCl. The level of NO was reduced in the serum and heart of HSD rats. NO donor sodium nitroprusside (SNP) reversed the increases of cleaved-caspase 3/caspase 3, cleaved-caspase 8/caspase 8, Bax/Bcl2 induced by NaCl (100 mM) in H9C2 cells and NRCM. SNP treatment attenuated the increases of cleaved-caspase 3/caspase 3, Bax/Bcl2, LC3 II/LC3 I, Beclin-1, and ATG7 in the heart, but had no effect on the blood pressure of HSD rats with HR. These results demonstrated that HSD enhanced cardiac damage independently of blood pressure. Exogenous NO supplementarity could alleviate the high salt–induced apoptosis and autophagy in cardiomyocytes.

## Introduction

Epidemiological studies have verified an association between high salt intake and cardiovascular diseases (Malta et al., 2018; Neupane et al., 2020; Xue et al., 2020). Clinical trials have demonstrated that reduced sodium intake was associated with a decrease in blood pressure, forming the basis for guideline thresholds (Dorsch et al., 2018; Humalda et al., 2020; Xu et al., 2020). Salt sensitivity is defined as the association between sodium intake and the increased risk of cardiovascular diseases in certain populations or individuals. Sodium intake may have differing effects on the individual’s cardiovascular outcomes like stroke, myocardial infarction, cardiovascular death, and congestive heart failure (O’Donnell et al., 2011; Sadanaga et al., 2019; Welsh et al., 2019).

High salt intake may cause cardiac damage, as indicated by increased cardiac interstitial and perivascular fibrosis, cardiomyocyte size, interventricular septum thickness, and left ventricular (LV) end-diastolic dimension and decreased LV fractional shortening (Komaki et al., 2018). Cardiac apoptosis and fibrosis were found to accelerate in rats with a high-salt diet (HSD) (Chang et al., 2019). High sodium intake increases blood pressure and promotes left ventricular hypertrophy in an independent manner (Le Corvoisier et al., 2010; Cai et al., 2020). However, whether high salt intake can cause cardiac damage independent of blood pressure is not well known.

Enhancing endothelial nitric oxide synthase (eNOS) function by restoring its coupling and subsequently reducing oxidative stress with folic acid may be a novel therapeutic approach to attenuate doxorubicin-induced cardiomyopathy (Octavia et al., 2017). Calcitonin gene-related peptide could protect cardiomyocytes against hypoxia-induced inflammation and apoptosis via nitric oxide (NO)–mediated pathway (Duan et al., 2016). However, whether NO attenuates high salt-induced apoptosis of cardiomyocytes is unclear. This study was to determine the high salt–induced cardiac damage which was independent of blood pressure, and explore whether NO could protect the cardiomyocytes from high salt–induced apoptosis and autophagy independent of blood pressure.

## Materials and Methods

### Animals

The experiments were carried out using male Sprague–Dawley (SD) rats weighing 160–180 g (Vital River Biological, Beijing, China). Approved by the Experimental Animal Care and Use Committee of Nanjing Medical University, all procedures were conducted in accordance with the Guide for the Care and Use of Laboratory Animals (NIH publication No. 85-23, revised in 1996). The rats were kept in a temperature-controlled room on a 12-h light–dark cycle with free access to standard chow and tap water. The study was approved by the ethics committee of Nanjing Medical University (No. 2007027)

### Rats Fed on HSD

Male SD rats weighing 160–180 g were randomly assigned to control diet (CD, 0.4% NaCl) group and HSD (8% NaCl; Research Diets, NJ, United States) group for 8 weeks. The rats with systolic blood pressure (SBP) ≥ 150 mm Hg were referred as hypertension (HTN) rats, rats with SBP between 130 and 150 mm Hg as hypertension-prone (HP) rats, and rats with SBP <130 mm Hg as hypertension-resistant (HR) rats. Rats were anesthetized with isoflurane (3.5%) and killed after a HSD feeding of 8 weeks. The hearts were removed and immediately frozen in liquid nitrogen and stored at −70°C until use.

### Measurement of Tail Artery Blood Pressure

Blood pressure was measured weekly in the tails of conscious rats using a non-invasive computerized tail-cuff system (Kent Scientific, CT, United States). The rats were warmed at 28°C for 10–20 min before the measurement, allowing tail arterial and steady pulsation. To minimize stress-induced fluctuations, the rats were pre-trained to get accustomed to daily blood pressure measurement for at least 1 week. The SBP, diastolic blood pressure (DBP), and mean artery pressure (MAP) of the tail artery were set as the average of at least 10 measurements (Li et al., 2013).

### Radiotelemetry Implantation and Data Acquisition

The rats were implanted with telemetry devices (Data Sciences International, MN, United States) through the abdominal aorta as described previously (Tazumi et al., 2016). Simply, the rats were placed on a heated surgical field in dorsal recumbency under anesthesia with isoflurane (2.5–3.5%). A midline incision in abdomen was made, and the abdominal aorta was isolated. The rat was implanted with a telemetry device, with the catheter inserted into the abdominal aorta and the transmitter implanted intraperitoneally. Arterial blood pressure was remotely monitored using a commercially available radiotelemetry data acquisition program (Ponemah v6.1; Data Sciences International).

### Echocardiography

Transthoracic echocardiography was performed under isoflurane anesthesia (2.5–3.5%) using an ultrasound system (Vevo 2100; VisualSonics, Toronto, Canada) with a 21-MHz probe. The ejection fraction (EF) and fractional shortening (FS) of the left ventricular (LV) were calculated. The LV internal diameter at diastolic (LVIDd), LV internal diameter at systolic (LVIDs), LV volumes in diastole (LVVd), LV volumes in systole (LVVs), LV end-diastolic anterior wall thickness (LVAWd), LV end-systolic anterior wall thickness (LVAWs), LV end-diastolic posterior wall thickness (LVPWd), and LV end-systolic posterior wall thickness (LVPWs) were measured. Measurements over three consecutive cardiac cycles were averaged.

### Rats Treated With Sodium Nitroprusside

The CD rats and HSD rats with HR (SBP < 130 mm Hg) were injected with NO donor sodium nitroprusside (SNP, Sigma, MO, United States). Rat received SNP (2.5 or 25 μg/kg) every 2 days for 2 weeks through the telemetry devices. Then the rats were anesthetized with isoflurane (2.5%) and killed after receiving SNP for 2 weeks. The hearts were removed, immediately frozen in liquid nitrogen, and stored at −70°C.

### Culturing of H9C2 Cells

The rat myoblast cell line H9C2 was cultured in supplemented Dulbecco’s Modified Eagle’s Medium (DMEM; Biochannel Biotechnology, Nanjing, China) supplemented with 10% fetal bovine serum (Biochannel Biotechnology), 100 U/ml of penicillin, and 100 μg/ml of streptomycin for 48 h at 37°C in 5% CO_2_ and 95% air.

### Grouping of H9C2 Cells

The H9C2 cells were divided into phosphate-buffered saline (PBS; Biochannel Biotechnology) group, mannitol (100 mM; Sigma, MO, United States) group, and NaCl groups of three doses (25, 50, or 100 mM) of NaCl. The cells were treated with mannitol or NaCl for 24 h. In another experiment, the H9C2 cells were divided into PBS, mannitol, NaCl, and NO donor SNP + NaCl groups. All cells were treated for 24 h.

### Culturing of Cardiomyocytes Isolated From Neonatal Rats

Primary cardiomyocytes were isolated from 1- to 2-day-old newborn SD rats (Vital River Biological, Beijing, China). Hearts were excised and digested through agitations in buffer containing collagenase type II (Worthington Biochemical Corporation, NJ, United States) and pancreatin (Sigma). The atria and great vessels were discarded. The ventricles were cut into small pieces and digested with collagenase type II and pancreatin. Cells from digestion were collected, cultured in complete DMEM (Biochannel Biotechnology) for 2–4 h to reduce fibroblasts, and enriched for cardiomyocytes. The cardiomyocytes were cultured at 37°C with 5% CO_2_ and 95% air.

### Western Blotting

Left ventricular tissues or cultured cells were sonicated in RIPA lysis buffer and homogenized. The debris was removed and the supernatant was obtained after centrifugation at 12,000 *g* for 10 min at 4°C. About 30–50 μg of proteins was loaded for electrophoresis, and probed with primary antibodies against caspase 3 (1:1000, #14220; Cell Signaling Technology, MA, United States), cleaved-caspase 3 (1:1000, #9664; Cell Signaling Technology), caspase 8 (1:1000, #4790; Cell Signaling Technology), cleaved-caspase 8 (1:1000 #8592; Cell Signaling Technology), Bax (1:1000, #5023; Cell Signaling Technology), Bcl2 (1:1000, #15071; Cell Signaling Technology), LC3 (1:1000, #12741; Cell Signaling Technology), eNOS (1:1000, #32027; Cell Signaling Technology), and p-eNOS (Ser1177; 1:1000, abs139915; Absin Bioscience, Shanghai, China) on PVDF membrane. GAPDH (1:1000, AF0006; Beyotime Biotechnology, Shanghai, China) was used as internal control. Images were analyzed using the Image-Pro Plus software.

### qRT-PCR

RNA was isolated from LV tissues or cultured cells using Trizol (Invitrogen). Total RNA (0.5 μg) was reversely transcribed into cDNA. qRT-PCR was performed using an ABI Prism 7900 sequencer (Applied Biosystems, Foster City, CA, United States). The primers are shown in Table 1. The relative level of mRNA expression was expressed as 2^–ΔΔ^ ^Ct^.

**TABLE 1:** List of utilized primers for qRT-PCR.

Gene	Species	Forward primer	Reverse primer
Beclin-1	Rat	CCATGCAG GTGAGCTTCGT	GAATCTGCGAGA GACACCATC
ATG7	Rat	GTTCGCCCCC TTTAATAGTGC	TGAACTCCAACGT CAAGCGG
iNOS	Rat	GTTCTCAGCCCA ACAATACAAGA	GTGGACGGGT CGATGTCAC
GAPDH	Rat	AGGTCGGTGTG AACGGATTTG	TGTAGACCATG TAGTTGAGGTCA

### TUNEL Staining

After treatment, the rate of apoptosis was detected in cells or LV tissues with a One Step TUNEL Apoptosis Assay Kit (C1089; Beyotime Biotechnology, Wuhan, China) following the manufacturer’s instructions. Briefly, having been rinsed with PBS for three times and then fixed with 4% PFA for 15 min, the heart sections or cells were permeabilized with 0.5% Triton X-100 for 5 min. The slides or cells were washed with PBS then incubated with TUNEL reaction mixture containing terminal deoxynucleotidyl transferase (TdT)–mediated cyanine 3 (Cy3)–labeled dUTP under humidified atmosphere for 60 min at 37°C in the dark. The negative and positive controls were carried out at the same time. After that, the nuclei were stained with DAPI for 5 min in the dark. The stained cells or slides were then observed at 570 nm under a confocal laser scanning microscope (Carl Zeiss, Oberkochen, Germany). Five fields were randomly selected in each slide or wall, and analyzed using ImageJ software in a blinded manner. The number of TUNEL-positive nuclei relative to the total number of nuclei was considered as a surrogate for apoptosis.

### Statistical Analyses

Data were presented as mean ± SEM. Using GraphPad Prism 7.0 (GraphPad Software, CA, United States), statistical significance among multiple groups was evaluated by one-way ANOVA with the Bonferroni *post hoc* test. A two-tailed *p*-value < 0.05 was considered statistically significant.

## Results

### Incidence of Non-hypertension and Hypertension

In 115 HSD-fed rats were 43 Non-HTN rats (37.39%) and 72 HTN rats (62.61%). Among the 43 Non-HTN rats, 18 (41.86%) were HP rats and 25 rats (58.14%) had normal blood pressure (Table 2).

**TABLE 2:** Incidence of non-hypertension and hypertension of rats with HSD.

Group	*n*	%
Total (CD + HSD)	135	
CD	20	14.81 (%Total)
HSD (HR + HP + HTN)	115	85.19 (%Total)
Non-HTN (HR + HP)	43	31.85 (%Total), 37.39 (%HSD)
HR	25	18.52 (%Total), 21.74 (%HSD), 58.14 (%Non-HTN)
HP	18	13.33 (%Total), 15.65 (%HSD), 41.86 (%Non-HTN)
HTN	72	53.33 (%Total), 62.61 (%HSD)

*CD, control diet; HSD, high salt diet; HR, hypertension-resistant; HP, hypertension-prone; HTN, hypertension; Non-HTN, non-hypertension.*

### Effects of HSD on Body Weight, Food Intake, Blood Pressure, and Cardiac Function

**FIGURE 1:**
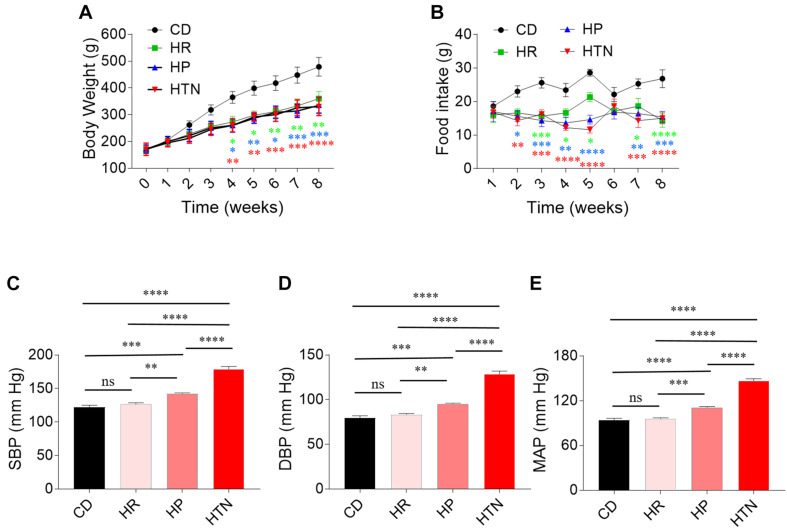
Effects of high-salt diet (HSD) on body weight and blood pressure in rats. **(A)** The body weight in the HSD groups with hypertension-resistant (HR), hypertension-prone (HP), and hypertension (HTN) was less than that in the control diet (CD) group. **(B–D)** The SBP, DBP, and MAP in HTN were higher than that in CD, HR, and HP groups. The results are expressed as mean ± SEM; *n* = 10 in CD group, *n* = 14 in HR and HP group, and *n* = 17 in HTN group. ^∗^*p* < 0.05, ^∗∗^*p* < 0.01, ^∗∗∗^*p* < 0.001, and ^****^p < 0.0001.

The body weight of rats in HSD groups with HR, HP, and HTN was less than that in CD group (Figure 1A). Food intake was lower in the three HSD groups than in CD groups (Figure 1B). The SBP, DBP, and MAP in HP and HTN groups were higher than those in CD and HR groups. The SBP, DBP, and MAP in HTN group was higher than those in other three groups (Figure 1). There was no difference in EF and FS between HR, HP, or HTN and CD groups. LVIDd, LVIDs, LVVd, and LVVs were slightly increased in HSD rats with HR, HP, and HTN, but did not show a significant difference. LVAWd, LVAWs, LVPWd, and LVPWs were higher in HSD rats with HR, HP, and HTN than in CD rats (Table 3).

**TABLE 3:** Cardiac function of rats with CD or HSD.

Variables	CD	HR	HP	HTN
EF (%)	72.262.05	70.601.33	69.882.13	69.881.72
FS (%)	42.921.84	41.621.21	41.441.86	41.421.41
LVIDd (mm)	7.990.25	8.180.25	8.760.14	8.760.21
LVIDs (mm)	4.620.19	4.760.08	5.130.19	5.140.19
LVVd (μl)	345.7122.56	384.4020.72	422.6315.13	424.4422.82
LVVs (μl)	112.778.49	115.724.18	127.7510.26	128.3511.62
LVAWd (mm)	1.620.06	2.010.07*	1.860.06*	1.990.11*
LVAWs (mm)	2.860.04	3.350.11*	3.340.14*	3.400.14*
LVPWd (mm)	1.680.05	2.200.11*	2.290.09*	2.150.06*
LVPWs (mm)	2.720.06	3.010.12*	3.180.10*	3.160.07*

CD, control diet; HSD, high salt diet; HR, hypertension-resistant; HP, hypertension-prone; HTN, hypertension.*p < 0.05 vs. CD group.

### Effects of HSD on Apoptosis and Autophagy in the Rat Heart Tissues

**FIGURE 2:**
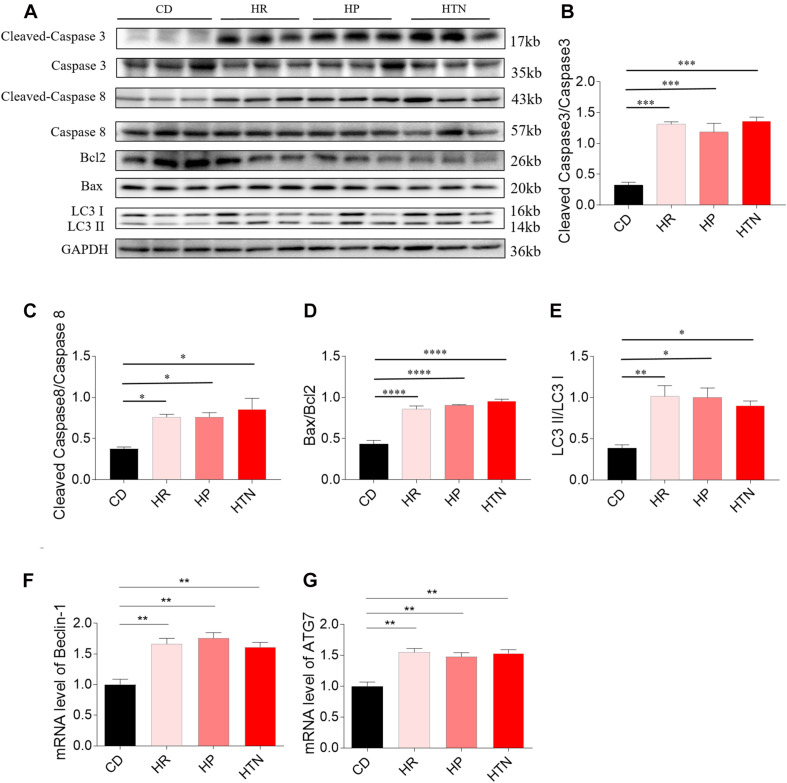
Effects of high-salt diet (HSD) on apoptosis and autophagy in the heart of hypertension-resistant (HR), hypertension-prone (HP), and hypertension (HTN) rats. The expression levels of cleaved-caspase 3/caspase 3 **(A,B)**, cleaved-caspase 8/caspase 8 **(A,C)**, Bax/Bcl2 **(A,D)**, LC3 II/LC3 I **(A,E)**, Beclin-1 **(F)** and autophagy related 7 (ATG7) **(G)** were increased in the heart of HSD rats with HR, HP and HTN. The results are expressed as mean ± SEM; *n* = 6 in each group. *p < 0.05, **p < 0.01, ***p < 0.001, and ****p < 0.0001.

The expression of cleaved-caspase 3/caspase 3 in the heart of HR, HP, and HTN rats fed with HSD was higher than that of CD rats. The level of cleaved-caspase 8/caspase 8 in the heart of HR, HP, and HTN rats fed with HSD was increased, compared with that in CD rats. The expression of Bax/Bcl2 in the heart of HR, HP, and HTN rats fed with HSD was significantly elevated. LC3 II/LC3 I expression level was higher in the heart of HR, HP, and HTN rats. The mRNA levels of Beclin-1 and ATG7 in the heart were increased in the three HSD groups. There were no significant differences in cleaved-caspase 3/caspase 3, cleaved-caspase 8/caspase 8, Bax/Bcl2, LC3 II/LC3 I, Beclin-1, and ATG7 levels among three HSD groups with HR, HP, and HTN (Figure 2).

### Effects of NaCl on H9C2 Apoptosis and Autophagy

**FIGURE 3:**
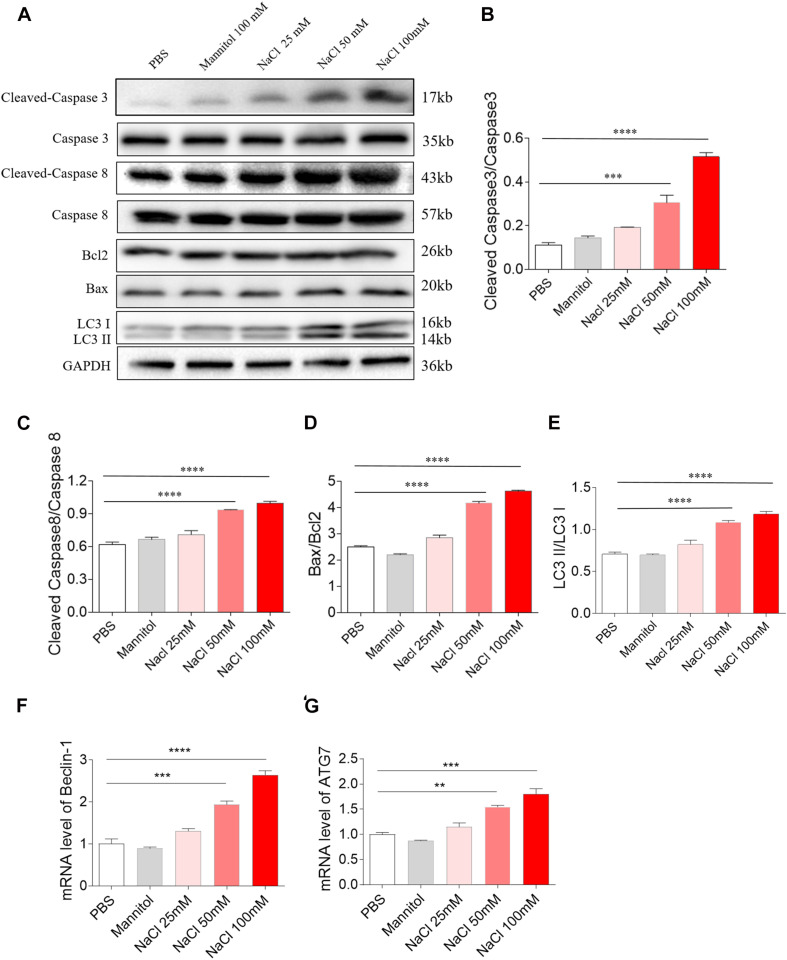
Effects of different doses of sodium chloride (NaCl) on apoptosis and autophagy in H9C2 cells. Middle (50 mM) and high (100 mM) doses of NaCl increased the levels of cleaved-caspase 3/caspase 3 **(A,B)**, cleaved-caspase 8/caspase 8 **(A,C)**, Bax/Bcl2 **(A,D)**, LC3 II/LC3 I **(A,E)**, Beclin-1 **(F)**, and autophagy related 7 (ATG7) **(G)** in H9C2 cells. The results are expressed as mean ± SEM; *n* = 6 in each group. **p < 0.01, ***p < 0.001, and ****p < 0.0001.

Middle (50 mM) and high (100 mM) doses of NaCl increased the level of cleaved-caspase 3/caspase 3 in H9C2 cells (Figures 3A,B). Middle (50 mM) and high (100 mM) doses of NaCl increased the levels of cleaved-caspase 8/caspase 8 in H9C2 cells (Figures 3A,C). The expression of Bax/Bcl2 was also increased in H9C2 cells by treatment with middle (50 mM) and high (100 mM) doses of NaCl (Figures 3A,D). LC3 II/LC3 I expression level was elevated in H9C2 cells by middle (50 mM) and high (100 mM) doses of NaCl (Figures 3A,E). The mRNA expression levels of Beclin-1 and ATG7 expression were increased in H9C2 cells by treating with middle (50 mM) and high (100 mM) doses of NaCl (Figures 3F,G). In H9C2 cells, 25 mM NaCl and mannitol (100 mM) exerted no effect on the levels of cleaved-caspase 3/caspase 3, cleaved-caspase 8/caspase 8, Bax/Bcl2, LC3 II/LC3 I, Beclin-1, and ATG7 compared with PBS (Figure 3).

### Effects of NaCl on eNOS, p-eNOS, iNOS, and NO Levels

**FIGURE 4:**
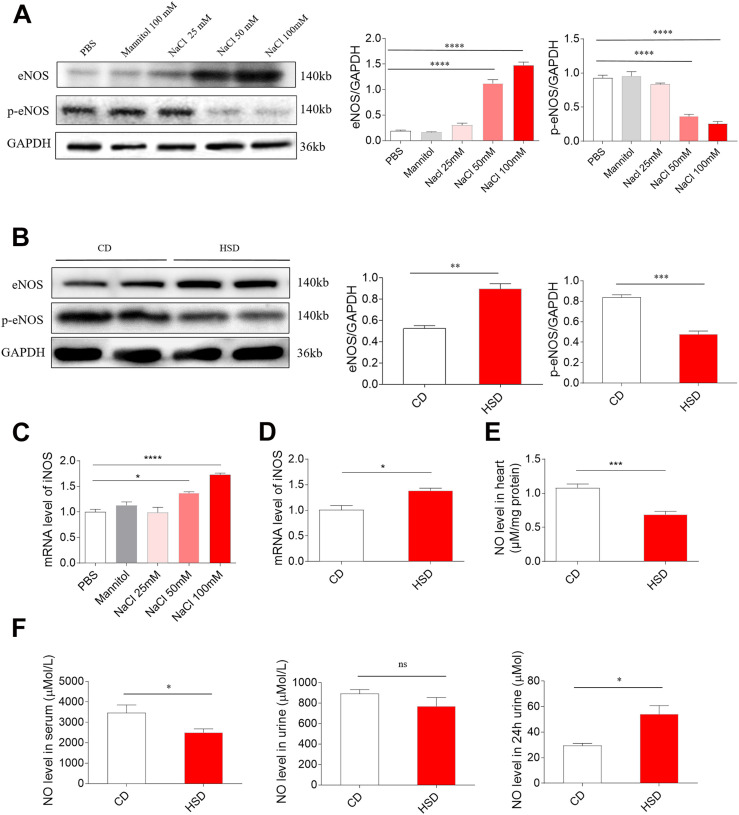
Levels of endothelial nitric oxide synthase (eNOS), p-eNOS, inducible NOS (iNOS), and nitric oxide (NO). **(A)** The level of eNOS was higher, but p-eNOS was lower in H9C2 cells treated with middle (50 mM) and high (100 mM) doses of sodium chloride (NaCl). **(B)** The expression of eNOS was increased, but p-eNOS was reduced in the heart of high-salt diet (HSD) rats with hypertension-resistant (HR). **(C)** The level of iNOS was higher in H9C2 cells treated with middle (50 mM) and high (100 mM) doses of NaCl. **(D)** The level of iNOS was increased in the heart of HSD rats with HR. **(E)** NO level was reduced in the heart of HSD rats with HR. **(F)** NO level was reduced in the serum, but was increased in the 24-h urine of HSD rats with HR. The results are expressed as mean ± SEM. *p < 0.05, **p < 0.01, ***p < 0.001, and ****p < 0.0001.

Middle (50 mM) and high (100 mM) doses of NaCl increased the level of eNOS in H9C2 cells, while low dose of NaCl (25 mM) and mannitol (100 mM) had no effect on the levels of eNOS in H9C2 cells. Middle (50 mM) and high (100 mM) doses of NaCl reduced p-eNOS level in H9C2 cells (Figure 4A). The level of eNOS was elevated, but p-eNOS level was reduced in the heart of HSD rats with HR compared with the control rats (Figure 4B). Middle (50 mM) and high (100 mM) doses of NaCl increased the mRNA level of iNOS in H9C2 cells, while low dose of NaCl (25 Mm) and mannitol (100 mM) had no effect on the mRNA levels of iNOS in H9C2 cells (Figure 4C). The mRNA level of iNOS was higher in the heart of HSD rats with HR compared with the CD rats (Figure 4D). The level of NO was lower in the heart of HSD rats with HR compared with the CD rats (Figure 4E). The NO level was reduced in the serum of HSD rats with HR. There was no significant difference in serum NO concentration between HSD rats with HR and CD rats. The NO level in the 24-h urine was reduced in the HSD rats (Figure 4F).

### Dose Effects of SNP on NaCl-Induced Apoptosis and Autophagy in H9C2 Cells

**FIGURE 5:**
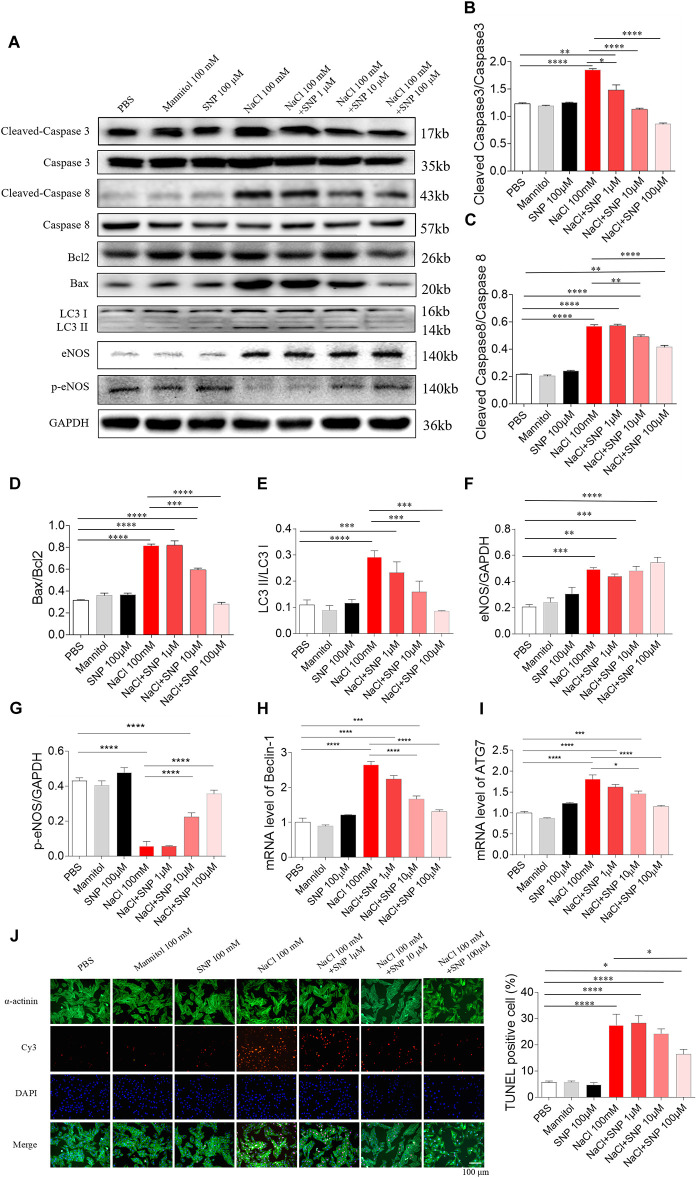
Effects of different doses of nitric oxide donor sodium nitroprusside (SNP) on NaCl-induced apoptosis and autophagy in H9C2 cells. **(A–I)**, SNP attenuated the increases in the levels of cleaved-caspase 3/caspase 3, cleaved-caspase 8/caspase 8, Bax/Bcl2, LC3 II/LC3 I, Beclin-1, and autophagy related 7 (ATG7), and enhanced the decrease of p-endothelial nitric oxide synthase (eNOS) induced by NaCl (100 mM) in H9C2 cells. **(J)** The increase of TUNEL-positive cell number was inhibited by high dose of SNP (100 μM), but not middle (10 μM) or low dose (1 μM) of SNP. The results are expressed as mean ± SEM. *p < 0.05, **p < 0.01, ***p < 0.001, and ****p < 0.0001.

All the three doses of SNP (1, 10, and 100 μM) attenuated the increases of cleaved-caspase 3/caspase 3 induced by NaCl (100 mM) in H9C2 cells. SNP (1 μM) partly blocked the NaCl-induced increase in cleaved-caspase 3/caspase 3 level in H9C2 cells. Middle (10 μM) and high (100 μM) doses of SNP completely abolished the NaCl-induced increase in the cleaved-caspase 3/caspase 3 level in H9C2 cells (Figures 5A,B). Middle (10 μM) and high (100 μM) doses of SNP partly blocked the NaCl-induced increase in cleaved-caspase 8/caspase 8 level in H9C2 cells, while SNP (1 μM) had no effect on the levels of cleaved-caspase 8/caspase 8 in H9C2 cells (Figures 5A,C). SNP (1 μM) exerted no effect on the Bax/Bcl2 level, but SNP (10 μM) partly blocked the NaCl-induced increase in the Bax/Bcl2 level, and SNP (100 μM) completely abolished the NaCl-induced increase in the Bax/Bcl2 level in H9C2 cells (Figures 5A,D). The increase of LC3 II/LC3 I induced by NaCl was inhibited by SNP (10 and 100 μM) in H9C2 (Figures 5A,E). The level of eNOS was increased in the H9C2 cells treated with NaCl (Figures 5A,F). The level of p-eNOS was reduced in the H9C2 cells treated with NaCl, and this decrease was reversed by SNP (10 and 100 μM) (Figures 5A,G). The mRNA levels of Beclin-1 and ATG7 were increased by NaCl in H9C2, and these increases were attenuated by SNP (10 and 100 μM) (Figures 5H,I). The number of positive cells for TUNEL staining was increased in NaCl-treated H9C2, and this increase was inhibited by high dose of SNP (100 μM), but not middle (10 μM) or low dose (1 μM) of SNP (Figure 5J).

### Effects of SNP on Three Doses of NaCl-Induced H9C2 Apoptosis and Autophagy

**FIGURE 6:**
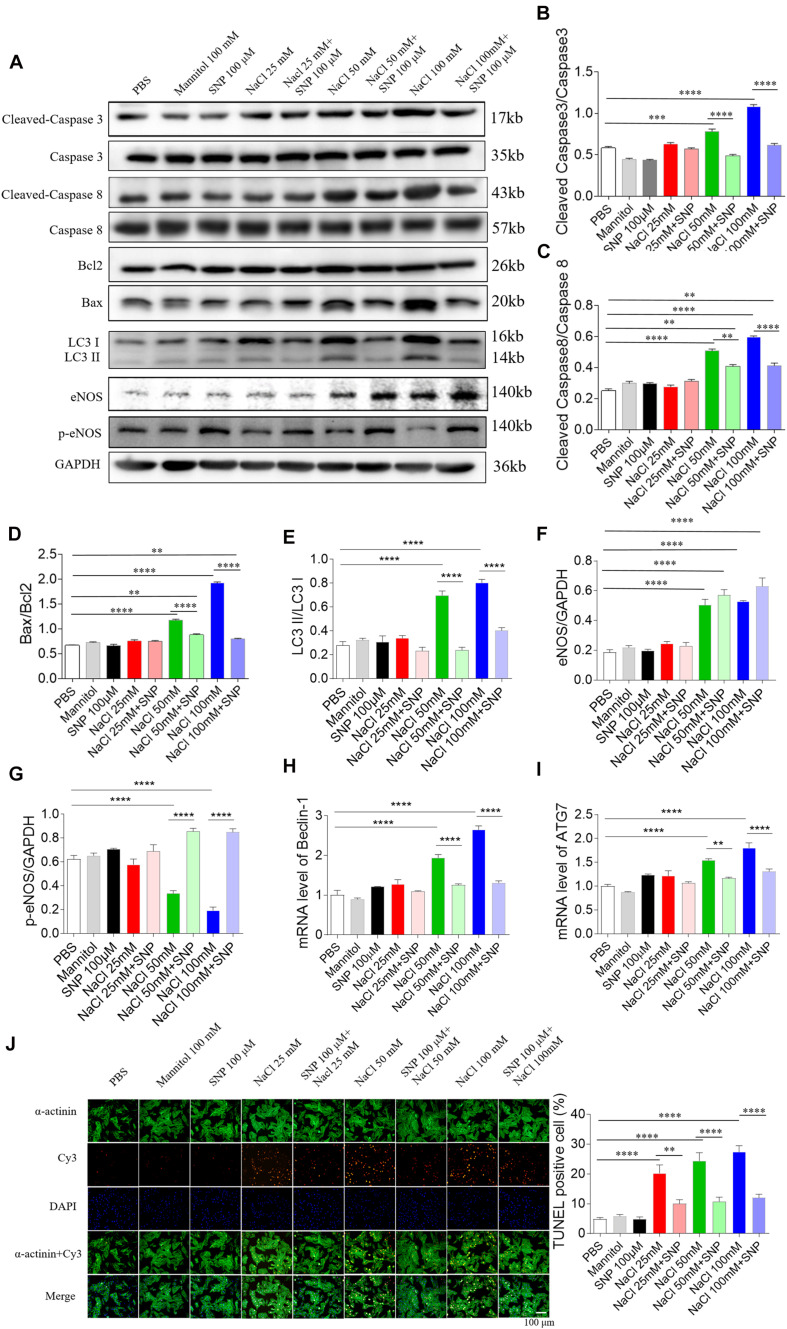
Effects of nitric oxide donor sodium nitroprusside (SNP) on apoptosis and autophagy induced by three doses of sodium chloride (NaCl) in H9C2 cells. **(A–I)** SNP (100 μM) attenuated the increases in the levels of cleaved-caspase 3/caspase 3, cleaved-caspase 8/caspase 8, and Bax/Bcl2 induced by NaCl (50 or 100 mM) in H9C2 cells. **(F)** The increases of TUNEL-positive cell numbers induced by three doses of NaCl in the H9C2 were attenuated by SNP (100 μM) treatment. The results are expressed as mean ± SEM. *p < 0.05, **p < 0.01, ***p < 0.001, and ****p < 0.0001.

In H9C2 cells, SNP (100 μM) treatment blocked the increases of cleaved-caspase 3/caspase 3 levels induced by two doses of NaCl (50 and 100 mM) (Figures 6A,B). The increased levels of cleaved-caspase 8/caspase 8 induced by NaCl (50 and 100 mM) were partly abolished by SNP (100 μM) in H9C2 cells (Figures 6A,C). The increases in the Bax/Bcl2 level induced by two doses of NaCl (50 and 100 mM) were completely blocked by SNP (100 μM) treatment in H9C2 cells (Figures 6A,D). The increase of LC3 II/LC3 I induced by NaCl (50 and 100 mM) was inhibited by SNP in H9C2 (Figures 6A,E). The levels of eNOS were increased in the H9C2 cells treated with NaCl (50 and 100 mM) (Figures 6A,F). The level of p-eNOS was reduced in H9C2 cells treated with NaCl (50 and 100 mM), and this decrease was reversed by SNP (Figures 6A,G). The increases of Beclin-1 and ATG7 induced by NaCl (50 and 100 mM) were inhibited by SNP in H9C2 (Figures 6H,I). The numbers of TUNEL-positive cells were increased in H9C2 treated with three doses of NaCl, and these increases were attenuated by SNP (100 μM) treatment (Figure 6J).

### Effects of SNP on Three Doses of NaCl-Induced Apoptosis and Autophagy of NRCM

**FIGURE 7:**
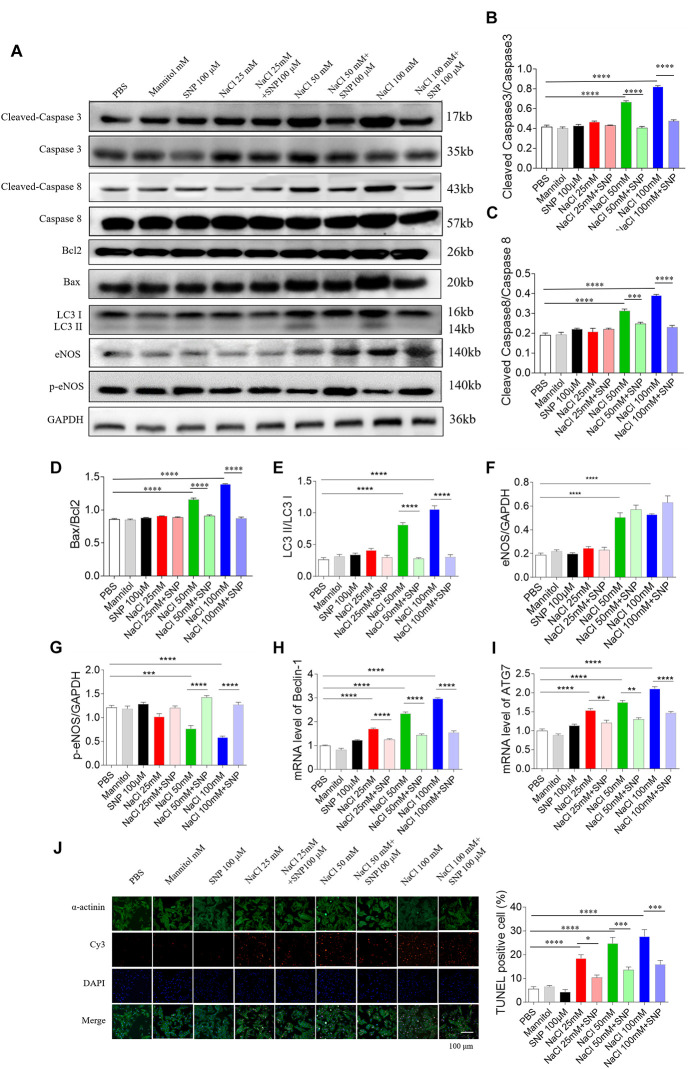
Effects of nitric oxide donor sodium nitroprusside (SNP) on apoptosis and autophagy induced by three doses of sodium chloride (NaCl) in primary neonatal rat cardiomyocytes (NRCM). SNP (100 μM) attenuated the increases in the levels of cleaved-caspase 3/caspase 3 **(A,B)**, cleaved-caspase 8/caspase 8 **(A,C)**, Bax/Bcl2 **(A,D)**, LC3 II/LC3 I **(A,E)**, Beclin-1 **(H)**, and autophagy related 7 (ATG7) **(I)**, and enhanced the decrease of p-endothelial nitric oxide synthase (eNOS) induced by NaCl (50 or 100 mM) in NRCM **(F,G)**. The increases of TUNEL-positive cell numbers induced by three doses of NaCl in the NRCM were attenuated by SNP (100 μM) treatment **(J)**. The results are expressed as mean ± SEM. *p < 0.05, **p < 0.01, ***p < 0.001, and ****p < 0.0001.

Sodium nitroprusside (100 μM) blocked the increase in cleaved-caspase 3/caspase 3 level induced by two doses of NaCl (50 and 100 mM) in NRCM (Figures 7A,B). The increased levels of cleaved-caspase 8/caspase 8 induced by NaCl (50 and 100 mM) were abolished by SNP (100 μM) treatment in NRCM (Figures 7A,C). The increases in the Bax/Bcl2 level induced by two doses of NaCl (50 and 100 mM) were inhibited by SNP (100 μM) in NRCM (Figures 7A,D). The expression level of LC3 II/LC3 I was increased in middle and high doses of NaCl-treated NRCM, then reversed by SNP (Figure 7E). The level of eNOS was increased in the NRCM treated with NaCl (50 and 100 mM) (Figures 7A,F). The level of p-eNOS was downregulated in NaCl-treated NRCM (50 and 100 mM), and this decrease was enhanced by SNP (Figures 7A,G). The mRNA levels of Beclin-1 and ATG7 were increased in NRCM treated with three doses of NaCl, and these increases were inhibited by SNP treatment (Figures 7H,I). The numbers of TUNEL-positive cells were increased in NRCM treated with three doses of NaCl, and these increases were attenuated by SNP (100 μM) treatment (Figure 7J).

### Effects of SNP on Apoptosis and Autophagy in the Heart of HSD Rats

**FIGURE 8:**
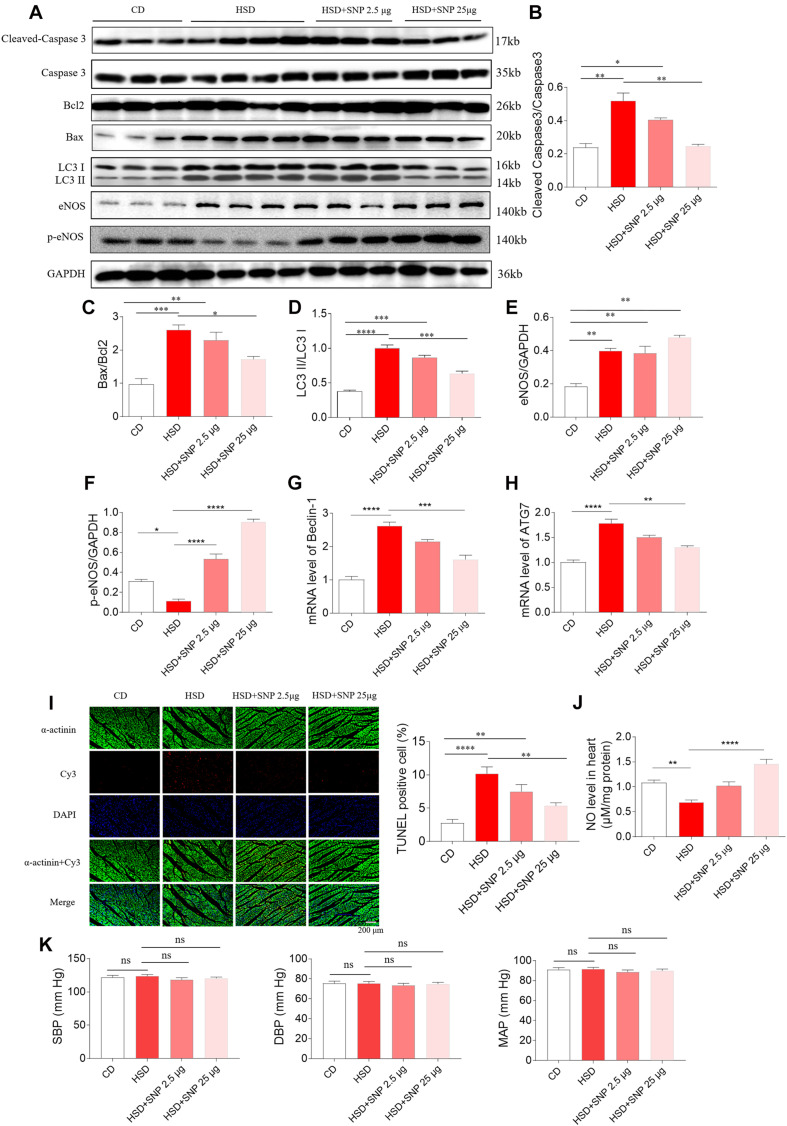
Effects of nitric oxide donor sodium nitroprusside (SNP) on HSD-induced apoptosis and autophagy in the heart of hypertension-resistant (HR) rats. **(A–H)** SNP attenuated the increases in cleaved-caspase 3/caspase 3, Bax/Bcl2, Beclin-1, and autophagy related 7 (ATG7), and enhanced the decrease of p-endothelial nitric oxide synthase (eNOS) induced by HSD in the heart of rats. **(I)** The increase of TUNEL-positive cell number was attenuated by SNP (25 μg/kg) treatment. **(J)** The decrease of nitric oxide (NO) in the heart of HSD rats with HR was increased by SNP administration. **(K)** SNP had no effects on the blood pressure of HR rats. The results are expressed as mean ± SEM. *n* = 6 for each group. *p < 0.05, **p < 0.01, ***p < 0.001, and ****p < 0.0001.

High-salt diet rats with HR were selected in the next study. HSD increased the levels of cleaved-caspase 3/caspase 3, Bax/Bcl2, and LC3 II/LC3 I in the rat heart. SNP (2.5 μg/kg) had no effects on the increases of cleaved-caspase 3/caspase 3, Bax/Bcl2, and LC3 II/LC3 I induced by HSD in the rat heart. SNP (25 μg/kg) inhibited the increases in cleaved-caspase 3/caspase 3, Bax/Bcl2, and LC3 II/LC3 I levels in the heart of HSD rats (Figures 8A–D). The level of eNOS was increased in the hearts of HSD rats (Figures 8A,E). The decrease of p-eNOS in the hearts of HSD rats was reversed by SNP (2.5 and 25 μg/kg) (Figures 8A,F). The increases of Beclin-1 and ATG7 m RNA were attenuated by SNP (25 μg/kg) (Figures 8G,H). The number of TUNEL-positive cardiomyocytes was increased in the hearts of HSD rats, and this increase was attenuated by SNP (25 μg/kg) treatment (Figure 8I). NO level was reduced in the heart of HSD rats, then increased by SNP (25 μg/kg) (Figure 8J). SNP had no effect on the blood pressure of HSD rats with HR (Figure 8K).

## Discussion

High-salt diet can aggravate cardiac damage, increase the expression of Bax, enhance the formation of reactive oxygen species, and initiate apoptosis in spontaneously hypertensive rats (Chao et al., 2016). Activating the eNOS/NO signaling cascades can protect from cardiac remodeling in rats after myocardial infarction (Li et al., 2017). The present study showed that high salt–induced apoptosis and autophagy in the rat heart and H9C2 cells could be attenuated by NO donor SNP.

Sudden death is associated with ventricular arrhythmias was observed in Dahl salt-sensitive rats fed with a HSD (Cho et al., 2018). High-salt intake disrupts the balance between oxidative stress and the antioxidant systems, leading to a higher risk of liver damage, fibrosis, and apoptosis (Wang et al., 2016). In the present study, HSD rats were divided into three groups according to blood pressure, including HR, HP, and HTN. We found that the levels of cleaved-caspase 3, cleaved-caspase 8, and Bax/Bcl2 were elevated in the heart of HSD rats with HR, HP, and HTN. Besides, cleaved-caspase 3, cleaved-caspase 8, and Bax/Bcl2 levels were increased in H9C2 cells treated with high salt. These results demonstrated that high salt promotes the apoptosis of cardiomyocytes independent of blood pressure.

High-level autophagy in the heart impairs cardiac function (Li et al., 2018; He et al., 2020). In Dahl rats fed with HSD, their lifespan is shortened by either hypertension-induced stroke or heart failure (Eisenberg et al., 2016). In the present study, LC3 II/LC3 I, Beclin-1, and ATG7 expression levels were higher in the NaCl-treated H9C2 cells. HSD increased the levels of LC3 II/LC3 I, Beclin-1, and ATG7 in the heart of rats with hypertension and non-hypertension. These results indicate that high salt boosts autophagy of cardiomyocytes independent of blood pressure, thus weakening cardiac function and increasing the risk of death.

Nitric oxide induces autophagy with AMP-activated protein kinase and protects the human dental pulp cells against NO-induced apoptosis (Park et al., 2017). The apoptosis is probably enhanced by NO overproduction and high oxidative stress in the heart of cirrhotic rats (Shafaroodi et al., 2017). However, NO can prevent the confluent endothelial monolayer from apoptosis (Lopez-Farre and Rodriguez-Feo, 1998). In the present study, the p-eNOS expression levels were reduced in NaCl-treated H9C2 cells and the heart of HSD rats. HSD decreased NO level in the serum and heart. NO donor SNP attenuated the increases of cleaved-caspase 3, cleaved-caspase 8, Bax/Bcl2, LC3 II/LC3 I, Beclin-1, and ATG7 in H9C2 cells induced by NaCl. Furthermore, the HSD-induced increases of cleaved-caspase 3, Bax/Bcl2, LC3 II/LC3 I, Beclin-1, and ATG7 in the heart of rats were suppressed after SNP treatment. These results indicate that eNOS level drops to decrease NO level. HSD decreases NO level to damage the heart, and upregulating eNOS/NO may be a strategy for treating cardiac damage caused by high salt.

The telemetry devices is used worldwide to continuously monitor blood pressure and electrocardiogram (Kiourti et al., 2014; Annoni et al., 2019). In our study, the rats receiving SNP treatment were implanted with telemetry devices in their abdominal aorta. We found that SNP had no effect on the blood pressure of non-hypertensive rats treated with HSD, which is supported by a previous study showing that SNP has no effect on blood pressure of normotensive rats (Hossain et al., 2018). These results indicated that NO attenuates the high salt–induced apoptosis and autophagy in the heart of HSD rats independently of blood pressure.

In addition to the mechanisms discussed previously, various complex molecular mechanisms, including ion channels, underlying excess salt intake–induced homeostasis imbalance, and subsequent hypertension and even cardiac damage are still not elucidated. Ion channels that present on the plasma membrane and intracellular organelles of all cells have been identified to play an irreplaceable role in regulating cardiac functions through secreting hormones and neurotransmitters, mediating vascular smooth muscle contraction, and supporting cellular integrity (Grant, 2009; Bartos et al., 2015).

Sufficient data confirmed the involvement of the epithelial sodium channel (ENaC), a trimeric ion channel, in the regulation of sodium reabsorption under high-salt intake conditions (Pavlov and Staruschenko, 2017). Diets rich in sodium, in combination with other factors, may harm the normal function of ENaC, contributing to hypertension and even cardiac dysfunction in Dahl salt-sensitive rats (Amin et al., 1979; Greene et al., 1990). Also, endogenous cardiac glycosides released by high-salt intake can inhibit Na(+), K(+)-ATPase activity, increasing Na+ on the submembrane of vascular smooth muscle cells, which may further facilitate Ca^2+^ entry and vasoconstriction, and eventually cause a corresponding increase in blood pressure (Iwamoto and Kita, 2006; Jaitovich and Bertorello, 2010). As a vital ion transport determining membrane potential (Iwamoto, 2006b), the Na(+)/Ca(2+) exchanger (NCX) type 1 was reported to increase vascular tone by mediating Ca^2+^ entry in arterial smooth muscle cells in response to excess salt intake, which could partly explain the link between dietary salt to salt-dependent hypertension (Iwamoto et al., 2005; Iwamoto, 2006a). Besides, a HSD could inhibit potassium channels (Oloyo et al., 2013). In addition, recent evidence showed that several transient receptor potential channels (TRP), including TRPV1 and TRPC3, are involved in high-salt intake–induced cardiac hypertrophy by mediating mitochondrial function via regulating mitochondrial calcium uptake (Lang et al., 2015; Ma et al., 2019). Thus, high-salt intake–mediated activation or inactivation of important ion channels in myocytes or vascular muscle cells could also be therapeutically useful for high-salt intake–induced hypertension and cardiac dysfunction, which remains to be further investigated in the future.

High-salt diet-induced hypertension has attracted attention. We currently found that HSD could cause cardiac damage in normotensive rats. This conclusion reminds us to pay close attention to salt intake before developing hypertension. However, the mechanism of high salt in cardiac damage was not well explored in the present study. We only found HSD enhanced apoptosis and autophagy of cardiomyocytes. In the future, we will employ other types of cells to analyze the effect of HSD on cardiac function.

In conclusion, HSD-induced cardiac damage in the heart of rats was independent of blood pressure, and this damage could be attenuated by NO. The eNOS/NO pathway might regulate HSD-induced apoptosis and autophagy in the rat heart.

## Data Availability Statement

The original contributions presented in the study are included in the article/supplementary material, further inquiries can be directed to the corresponding author/s.

## Ethics Statement

The animal study was reviewed and approved by the Experimental Animal Care and Use Committee of Nanjing Medical University.

## Author Contributions

YL designed and performed the experiments and prepared the manuscript. XW, YM, and CL assisted with experiments planning and execution. YW assisted with experiments. JT assisted in project conception and experimental design. KZ and PL designed the experiments, provided the experimental insight, and edited the manuscript. All the authors contributed to the article and approved the submitted version.

## Conflict of Interest

The authors declare that the research was conducted in the absence of any commercial or financial relationships that could be construed as a potential conflict of interest.
