# Corrigendum: Nitric oxide alleviated high salt-induced cardiomyocyte apoptosis and autophagy independent of blood pressure in rats

**DOI:** 10.3389/fcell.2024.1419893

**Published:** 2024-07-25

**Authors:** Yong Li, Xiaoguang Wu, Yukang Mao, Chi Liu, Yiting Wu, Junzhe Tang, Kun Zhao, Peng Li

**Affiliations:** ^1^ Department of Cardiology, The First Affiliated Hospital of Nanjing Medical University, Nanjing, China; ^2^ Department of Cardiology, The Affiliated Hospital of Xuzhou Medical University, Xuzhou, China; ^3^ The First School of Clinical Medicine, Nanjing Medical University, Nanjing, China

**Keywords:** high-salt diet, cardiomyocytes, apoptosis, autophagy, nitric oxide, sodium nitroprusside

In the published article, there was an error in Figure 5J as published. The wrong representative image of TUNEL-staining was used. The corrected Figure 5 and its caption appear below.

**FIGURE 5:**
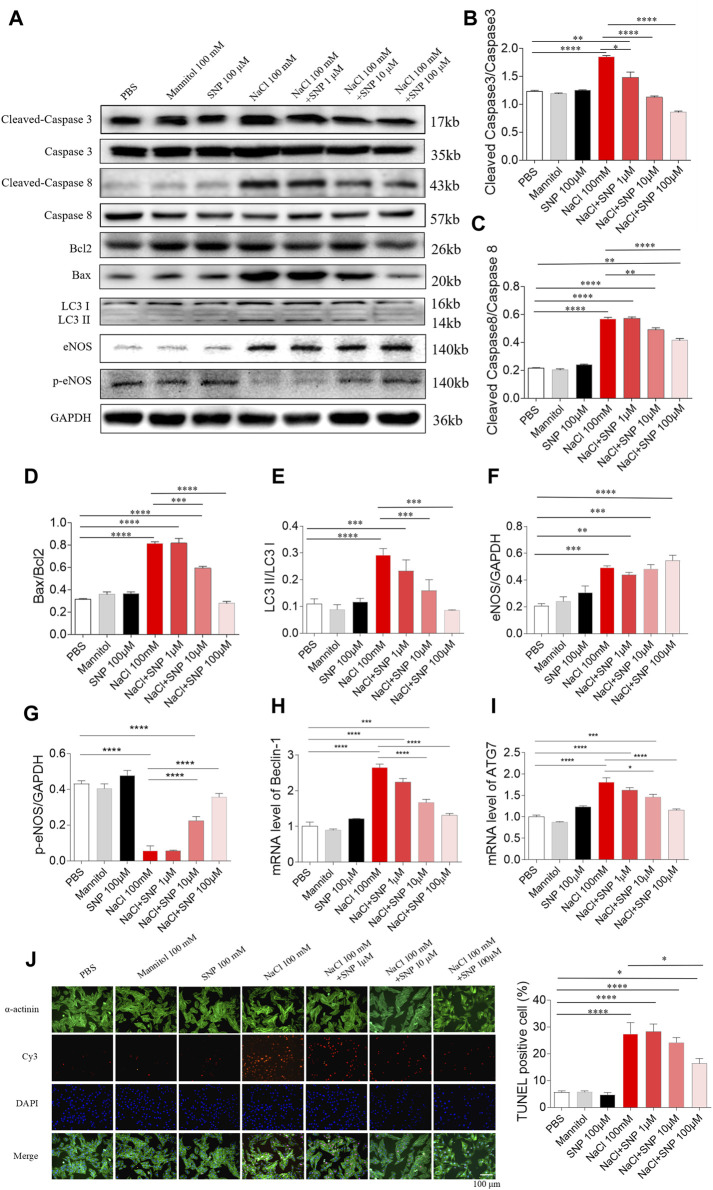
Effects of different doses of nitric oxide donor sodium nitroprusside (SNP) on NaCl-induced apoptosis and autophagy in H9C2 cells. **(A–I)**, SNP attenuated the increases in the levels of cleaved-caspase 3/caspase 3, cleaved-caspase 8/caspase 8, Bax/Bcl2, LC3 II/LC3 I, Beclin-1, and autophagy related 7 (ATG7), and enhanced the decrease of p-endothelial nitric oxide synthase (eNOS) induced by NaCl (100 mM) in H9C2 cells. **(J)** The increase of TUNEL-positive cell number was inhibited by high dose of SNP (100 µM), but not middle (10 µM) or low dose (1 µM) of SNP. The results are expressed as mean ± SEM. *p < 0.05, **p < 0.01, ***p < 0.001, and ****p < 0.0001.

In the published article, there was an error in Figure 7J as published. The wrong representative image of TUNEL-staining was used. The corrected Figure 7 and its caption appear below.

**FIGURE 7:**
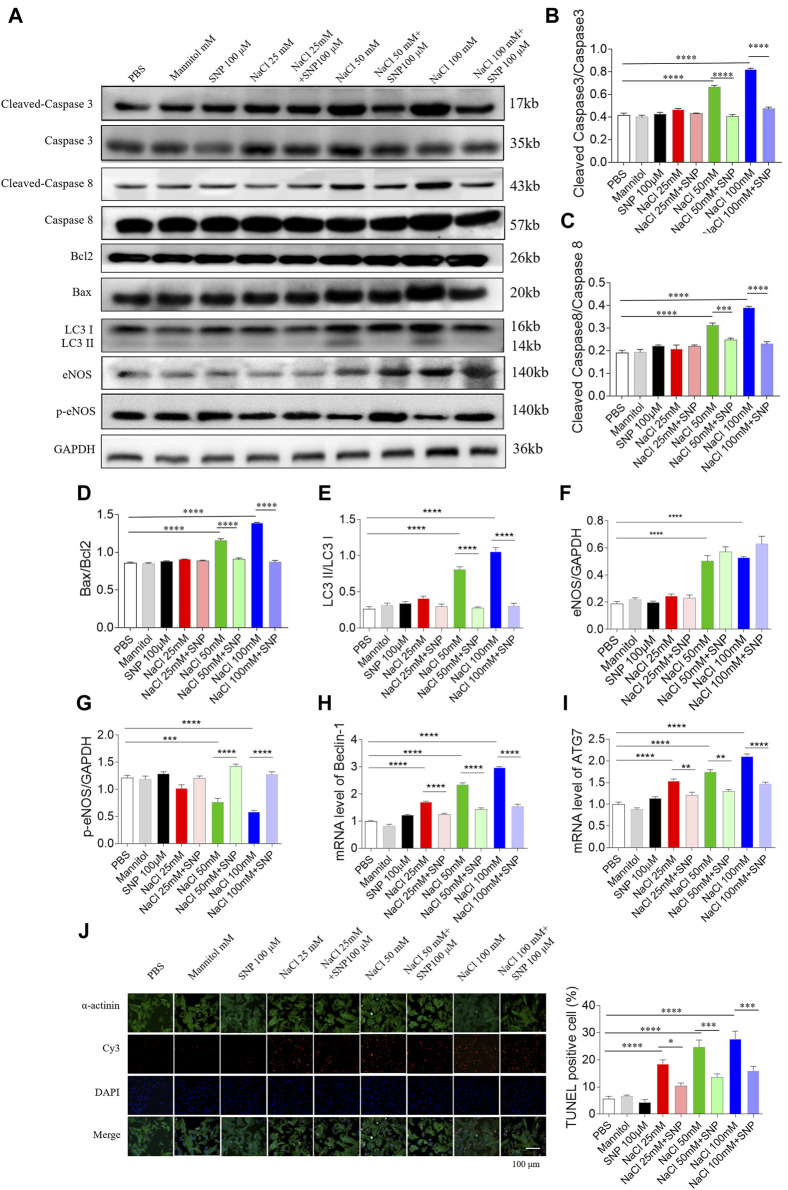
Effects of nitric oxide donor sodium nitroprusside (SNP) on apoptosis and autophagy induced by three doses of sodium chloride (NaCl) in primary neonatal rat cardiomyocytes (NRCM). SNP (100 µM) attenuated the increases in the levels of cleaved-caspase 3/caspase 3 **(A, B)**, cleaved-caspase 8/caspase 8 **(A, C)**, Bax/Bcl2 **(A, D)**, LC3 II/LC3 I **(A,E)**, Beclin-1 **(H)**, and autophagy related 7 (ATG7) **(I)**, and enhanced the decrease of p-endothelial nitric oxide synthase (eNOS) induced by NaCl (50 or 100 mM) in NRCM **(F, G)**. The increases of TUNEL-positive cell numbers induced by three doses of NaCl in the NRCM were attenuated by SNP (100 µM) treatment **(J)**. The results are expressed as mean ± SEM. *p < 0.05, **p < 0.01, ***p < 0.001, and ****p < 0.0001.

The authors apologize for these errors and state that this does not change the scientific conclusions of the article in any way. The original article has been updated.

